# Electrical Girl

**Published:** 1846-06

**Authors:** 


5. *Electrical Girl.*—M. Arago stated to the French Academy of Sciences, at their meeting on the 16th of Feb. last, that he had been called upon to witness some of the most singular phenomena which he had ever beheld, in the shape of electric discharges of the most violent character, proceeding from the person of a young girl, aged thirteen, lately submitted to his inspection. This truly remarkable child overturns tables and chairs by merely touching them with her apron. When she sits down, the moment her feet touch the ground, the chair is upset, and she is suddenly propelled with considerable force. M. Arago said he had seen all these experiments, and had not been able to detect any trick. He begged the Academy would appoint a committee to investigate the matter.—*Med. Times*, Feb. 1846.

